# Evaluation of ^131^I-Anti-Angiotensin II Type 1 Receptor Monoclonal Antibody as a Reporter for Hepatocellular Carcinoma

**DOI:** 10.1371/journal.pone.0085002

**Published:** 2014-01-08

**Authors:** Pan-Pan Hao, Yan-Ping Liu, Chang-Ya Yang, Ting Liang, Chao Zhang, Jing Song, Jian-Kui Han, Gui-Hua Hou

**Affiliations:** 1 Key Laboratory for Experimental Teratology of the Ministry of Education and Institute of Experimental Nuclear Medicine, School of Medicine, Shandong University, Jinan, China; 2 The Key Laboratory of Cardiovascular Remodeling and Function Research, Chinese Ministry of Education and Chinese Ministry of Health, Qilu Hospital, Shandong University, Jinan, China; 3 Department of Nuclear Medicine, Qilu Hospital, Shandong University, Jinan, China; National Cancer Institute, NIH, United States of America

## Abstract

**Background:**

Finding a specific agent is useful for early detection of tumor. Angiotensin II type 1 receptor (AT_1_R) was reported to be elevated in a variety of tumors and participate in tumor progression. The aim of our study was to evaluate whether ^131^I-anti-AT_1_R monoclonal antibody (mAb) is an efficient imaging reporter for the detection of hepatocellular carcinoma.

**Methodology/Principal Findings:**

AT_1_R mAb or isotype IgG was radioiodinated with ^131^I and the radiochemical purity and stability of the two imaging agents and the affinity of ^131^I-anti-AT_1_R mAb against AT_1_R were measured. 3.7 MBq ^131^I-anti-AT_1_R mAb or isotype ^131^I-IgG was intravenously injected to mice with hepatocellular carcinoma through tail vein, and then the whole-body autoradiography and biodistribution of the two imaging agents and the pharmacokinetics of ^131^I-anti-AT_1_R mAb were studied. ^131^I-anti-AT_1_R mAb and ^131^I-IgG were successfully radioiodinated and both maintained more stable in serum than in saline. The ^131^I-anti-AT_1_R mAb group showed much clearer whole-body images for observing hepatocellular carcinoma than the ^131^I-IgG group. The biodistributions of the two imaging agents suggested that hepatocellular carcinoma tissue uptook more ^131^I-anti-AT_1_R mAb than other tissues (%ID/g = 1.82±0.40 and T/NT ratio = 7.67±0.64 at 48 h), whereas hepatocellular carcinoma tissue did not selectively uptake ^131^I-IgG (%ID/g = 0.42±0.06 and T/NT ratio = 1.33±0.08 at 48 h). The pharmacokinetics of ^131^I-anti-AT_1_R mAb was in accordance with the two-compartment model, with a rapid distribution phase and a slow decline phase. These results were further verified by real-time RT-PCR, immunohistochemistry staining and Western blot.

**Conclusions/Significance:**

^131^I-anti-AT_1_R mAb may be a potential target for early detection of tumor.

## Introduction

Hepatocellular carcinoma is the sixth most prevalent cancer and the third most frequent cause of cancer-related death [1], [2]. Although biopsy is considered to be the gold standard for diagnosis of hepatocellular carcinoma, it is more invasive than serum biomarkers or imaging techniques. The detection of hepatocellular carcinoma mainly relies on serum alpha-fetoprotein and liver imaging techniques such as B-mode ultrasound, X-ray computed tomography, magnetic resonance imaging and positron emission computed tomography [3]–[5]. But when these abnormalities can be detected, the staging of hepatocelluar carcinoma usually goes into an advanced symptomatic stage [1]. Therefore, exploring a new non-invasive technology to detect hepatocellular carcinoma in a very early stage is urgently needed. Molecular imaging is such a technology, which integrates the principles of cell and molecular biology, immunology, nuclear medicine and diagnostic imaging [6]. Then it becomes an issue to find a target molecule which could specifically detect tumor in an early stage.

Overexpression of angiotensin II type 1 receptor (AT_1_R) in a variety of tumors has been reported recently [7]–[10]. AT_1_R promotes tumor growth and angiogenesis partially through upregulation of vascular endothelial growth factor (VEGF) [11], [12]. We hypothesized that AT_1_R expression might be upregulated in hepatocellular carcinoma tissue and ^131^I-anti-AT_1_R IgG monoclonal antibody (mAb) might be a new potential molecular imaging agent in tumor. The aim of this study was to validate this hypothesis.

## Materials and Methods

### Ethics statement

The animal protocol was reviewed and approved by the Institutional Animal Care and Use Committee at School of Medicine, Shandong University.

### Cell culture and reagents

Murine hepatocellular carcinoma cell line H22 (Cell Bank of Chinese Academy of Sciences, Shanghai, China), murine liver cell line NCTC clone 1469 (CCL-9.1, American Type Culture Collection, Manassas, VA), human cervical cancer cell line Hela (CCL-2, American Type Culture Collection) and rat adrenal pheochromocytoma cell line PC12 (CRL-1721, American Type Culture Collection) were cultured in RPMI 1640 medium (Invitrogen, Carlsbad, CA), all supplemented with 100 units·mL^−1^ penicillin (Gibco BRL, Gaithersburg, MD), 100 units·mL^−1^ streptomycin (Gibco BRL) and 10% fetal bovine serum (Gibco BRL) at 37°C in a 95% air/5% CO_2_ humidified atmosphere.

### Animal model

Male BALB/c mice (6–8 weeks old) were purchased from Shandong University Animal Center and were maintained under pathogen-free conditions. The BALB/c mice (36 per group) were injected subcutaneously with 1×10^7^ H22 cells in 0.1 mL phosphate buffered saline into the right upper back to establish a hepatoma model.

### Radioiodination of anti-AT_1_R mAb and isotype IgG

50 µg anti-AT_1_R mAb (Abcam, Cambridge, UK) or isotype IgG (Abcam) was iodinated with 15 µL Na^131^I (185 MBq) (China Institute of Atomic Energy, Beijing, China) using the Iodogen method as described previously [13]. Radioiodinated anti-AT_1_R mAb and isotype IgG were separated from free iodine using size exclusion columns (Sephadex G-25, Amersham Pharmacia Biotech, Uppsala, Sweden).

### Radiochemical purity and stability

Radiochemical purity was determined by paper chromatographic method using strips on two-paper sheet (1 cm width and 13 cm length) as described [14] with modifications. Briefly, an aliquot of 2 µL ^131^I-anti-AT_1_R mAb or ^131^I-IgG was added into 400 µL serum or saline. 2 µL of the mixture was placed 2 cm above the lower edge and was allowed to evaporate spontaneously, one strip was developed with acetone and the other was developed with ethanol: water: ammonium hydroxide mixture (2∶5∶1). After complete development, the paper sheet was removed, dried, and cut into strips of 1 cm width; and then each strip was counted in a well-type c-counter. The percentage of radiochemical yield was calculated as the ratio of the radioactivity of ^131^I-anti-AT_1_R mAb or ^131^I-IgG to the total activity multiplied by 100. Radiochemical purities were measured at 1, 6, 24, 48, 72 and 96 hours, respectively, to assess the stability.

### Radioligand-based binding assay

The radioligand-based binding assay was carried out in borosilicate glass tubes as described [15], [16] with modifications. For saturation studies, a reaction mixture contained 200 µL H22 cells (5×10^6^/mL) and 100 µL ^131^I-anti-AT_1_R mAb (0.1–32 nM, diluted in 1×PBS) in a final volume of 500 µL. 10^−1^–10^5^ nM unlabeled anti-AT_1_R mAb and 12 nM ^131^I-anti-AT_1_R mAb were used for competition binding assay. The mixture was incubated at 37°C for 2 h. The bound radioligand was separated by rapid vacuum filtration through Whatman GF/B filters using a cell harvester followed by 3×2 mL washes of PBS at room temperature. The radioactivity of filters containing the bound radioligand was assayed in test tube by Wipe Test/Well Counter (Caprac; Capintec, Ramsey, NJ). The results of saturation and inhibition experiments were subjected to nonlinear regression analysis and the equilibrium dissociation constant (K_D_), the maximum number of binding sites (B_max_), the inhibitor constant (K_i_) and the half maximal inhibitory concentration (IC_50_) were calculated.

### Whole-body autoradiography

Whole-body autoradiography was performed as described [17] with modifications. 10% potassium iodide was added to drinking water 3 days before injection of ^131^I-labeled antibody to block the thyroid gland. 12 days after injection of H22 cells, 3.7 MBq ^131^I-anti-AT_1_R mAb or ^131^I-IgG was respectively injected into the mice through tail vein. Whole-body autoradiography was performed at 1, 6, 24, 48 and 72 hours after injection, respectively. The anesthetized mice were fixed on the storage phosphor screen plate in supine position with four limbs stretched in order to make the tumor tightly close to the plate. The plate was exposed to a mouse for 15 minutes in a darkroom. After exposure, the plate was scanned by Cyclone Plus Storage Phosphor System (PerkinElmer) and analyzed using the OptiQuant Acquisition software.

### Biodistribution of ^131^I-anti-AT_1_R mAb and ^131^I-IgG

Six mice of each group were sacrificed at 1, 6, 24, 48 and 72 hours after injection, respectively, and blood, tumor, muscular tissue on the left side and main organs were removed, weighed, and counted radioactivity in the gamma counter. The percent injected dose per gram (%ID/g) and target to non-target ratio (T/NT) were calculated.

### Pharmacokinetic analysis

10 µL blood samples were taken from periorbital vein of six mice at 0, 1, 3, 6, 12, 24, 48, 72, 96 and 120 hours after injection of ^131^I-anti-AT_1_R mAb and then the radioactivity was measured by Liquid Scintillation Counting. The distribution half-life (T_1/2α_), the elimination half-life (T_1/2β_) and the mean residence time (MRT) were calculated.

### Real-time PCR

The primer for AT_1_R was chosen in cDNA portions by accessing mouse sequences in GenBank. The sequences were as follows: sense primer, 5′-GAAGAACAAGCCAAGAAATGATG-3′; antisense primer, 5′-TTGATGACTCCAGGTTAGCAGAT-3′ (887 bp). Total RNA was extracted from the cell lines or freshly isolated tumor, contralateral muscle and normal liver tissue by use of TriZol reagent (Invitrogen, Carlsbad, CA). Oligo (dT) primed cDNA synthesis involved use of Superscript™III reverse transcriptase (Invitrogen). Transcripts were amplified from reverse-transcribed cDNA by use of SYBR Green (Invitrogen). Cycling conditions for amplification were as follows: 4 min, denaturation step at 94°C; followed by 35 cycles of 30 s, at 94°C, 1 min, at 55°C, and 1 s, at 72°C. Quantitative assessment of relative gene expression levels involved the 2^−△△CT^ method.

### Histology

Tissues were fixed in phosphate-buffered 4% paraformaldehyde, embedded in paraffin, and cut into 4-µm thick sections. Sections were deparaffinized and stained with hematoxylin and eosin using a standard protocol to determine morphology. AT_1_R protein expression was determined by immunostaining with anti-AT_1_R mAb (1∶50) using the streptavidin-biotin method. Image-Pro Plus v5.0.2 (Media Cybernetics, Inc., Bethesda, MD) was used for quantitative assessment of relative AT_1_R protein expression levels.

### Western blot

SDS polyacrylamide gel electrophoresis and Western blot analysis were performed as described previously [18]. Membranes were incubated with anti-AT_1_R mAb (1∶400) or a primary antibody against β-actin (1∶1000; Cell Signaling Technology, Danvers, MA), followed by appropriate horseradish peroxidase-labeled secondary antibodies. Protein levels were normalized to that of β-actin as an internal control. HeLa cells and PC12 cells were used as positive controls.

### Statistical analysis

SPSS v11.5 (SPSS Inc., Chicago, IL) was used for statistical analysis. Continuous data were expressed as mean ± SEM and compared by one-way ANOVA, followed by unpaired t-test or paired t-test as appropriate. A *P* value <0.05 was considered statistically significant.

## Results and Discussion

### Radioiodination of anti-AT_1_R mAb and isotype IgG

^131^I-anti-AT_1_R mAb and ^131^I-IgG were successfully radioiodinated. The radiochemical purity of ^131^I-anti-AT_1_R mAb was 92.8% and that of ^131^I-IgG was 93.2%. The specific activity of ^131^I-anti-AT_1_R mAb was 35.24±5.76 MBq/µmol and that of ^131^I-IgG was 38.61±7.18 MBq/µmol.

### The affinity of ^131^I-AT_1_R mAb against AT_1_R

**Figure 1:**
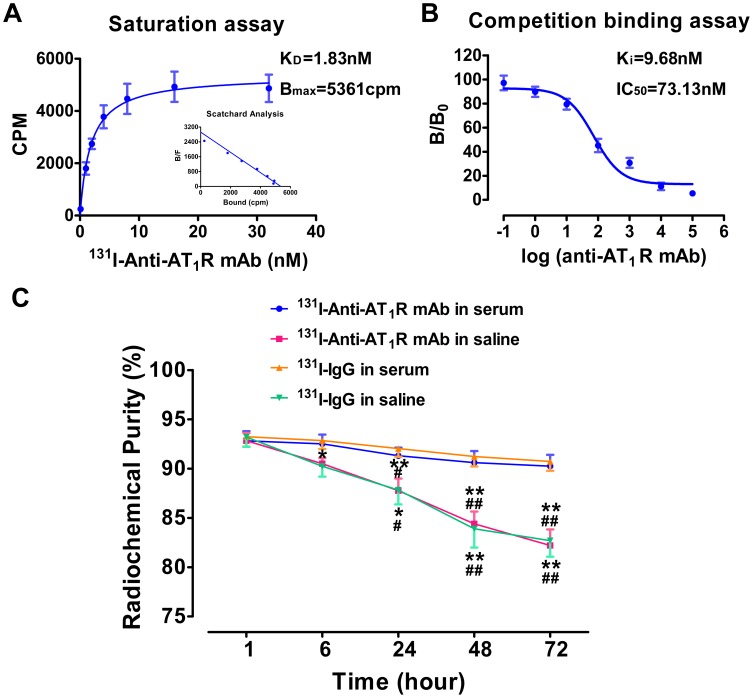
The affinity and stability of ^131^I-AT_1_R mAb and ^131^I-IgG. (A) The saturation assay of ^131^I-AT_1_R mAb. K_D_, equilibrium dissociation constant; B_max,_ maximum number of binding sites. (B) The competition binding assay of ^131^I-AT_1_R mAb. K_i_, inhibitor constant; IC_50_, half maximal inhibitory concentration. (C) The radiochemical purity of ^131^I-AT_1_R mAb and ^131^I-IgG. *P<0.05 and **P<0.01 vs. 1 hour; ^#^ *P*<0.05 and ^##^ *P*<0.01 vs. the same time-point in serum.

Radioligand-based binding assay is one of the most sensitive techniques available to quantitatively determine the affinity of one antibody against a certain receptor. Saturation assay showed that ^131^I-anti-AT_1_R mAb displayed saturable binding with H22 cells and K_D_ and B_max_ were 1.83±0.48 nM and 5361±345.3 cpm, respectively. Transformation of the saturation binding of ^131^I-anti-AT_1_R mAb to Scatchard plots gave linear plots, suggesting that it involved a single population of binding sites (Fig. 1A). Competitive binding assay was also conducted with ^131^I-anti-AT_1_R mAb as radioligand. The unlabeled anti-AT_1_R mAb competed effectively with ^131^I-anti-AT_1_R mAb binding sites on H22 cells at low micromole concentrations and K_i_ and IC_50_ were 9.68±1.33 nM and 73.13±1.33 nM, respectively (Fig. 1B). These results revealed high affinity of ^131^I-anti-AT_1_R mAb against AT_1_R.

### The stability of ^131^I-AT_1_R mAb and ^131^I-IgG

The radiochemical purities of ^131^I-anti-AT_1_R mAb and ^131^I-IgG were still over 90% in serum, and declined under 80% in saline at 72 hours, indicating that they maintained more stable in serum than in saline. There was no significant difference between the two imaging agents (Fig. 1C).

### The whole-body autoradiography in the hepatoma mice

**Figure 2:**
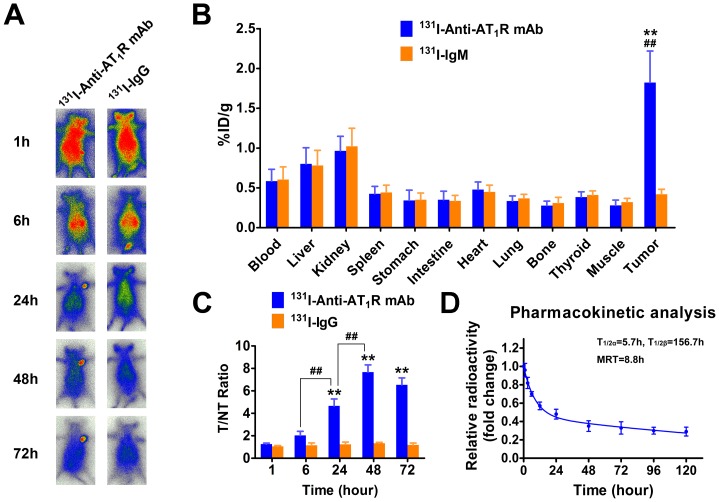
The whole-body autoradiography and biodistribution of ^131^I-anti-AT_1_R mAb and ^131^I-IgG in a mouse model of hepatocellular carcinoma. (A) The whole-body autoradiography. (B) The %ID/g of main tissues at 48 hours. **P<0.01 vs. muscle. ^##^ *P*<0.01 vs. ^131^I-IgG. (C) The T/NT ratios. **P<0.01 vs. 1 hour. ^##^ *P*<0.01. (D) The pharmacokinetics analysis of ^131^I-AT_1_R mAb. T_1/2α_, distribution half-life; T_1/2β_, elimination half-life; MRT, mean residence time.

The ^131^I-anti-AT_1_R mAb group showed much clearer whole-body images for observing hepatocellular carcinoma than the ^131^I-IgG group 24 hours after injection of radioiodinated anti-AT_1_R mAb or isotype IgG and the difference reached a peak at 48 hours (Fig. 2A). These data demonstrate that the ^131^I-anti-AT_1_R mAb appears to be more specific than ^131^I-IgG for targeting hepatocellular carcinoma.

### Biodistribution of ^131^I-anti-AT_1_R mAb and ^131^I-IgG

In the ^131^I-anti-AT_1_R mAb group, %ID/g of the tumor was higher than that of other tissues, and T/NT reached a peak at 48 hours after injection (%ID/g = 1.82±0.40 and T/NT ratio = 7.67±0.64). In the ^131^I-IgG group, there was no significant increase of %ID/g in the tumor and T/NT remained stable throughout the experiment (Fig. 2B and C). The results indicated that hepatocellular carcinoma tissue uptakes more ^131^I-anti-AT_1_R mAb than other tissues, whereas hepatocellular carcinoma tissue does not selectively uptake ^131^I-IgG. Thus, ^131^I-anti-AT_1_R mAb may be a potential imaging agent for targeting hepatocellular carcinoma.

### Pharmacokinetic analysis

Pharmacokinetic analysis showed that the pharmacokinetics of ^131^I-anti-AT_1_R mAb was in accordance with the two-compartment model, with a rapid distribution phase and a slow decline phase. T_1/2α_ and T_1/2β_ were 5.7 h and 156.7 h, respectively, and MRT was 8.8 h (Fig. 2D).

### AT_1_R mRNA and protein expression

**Figure 3:**
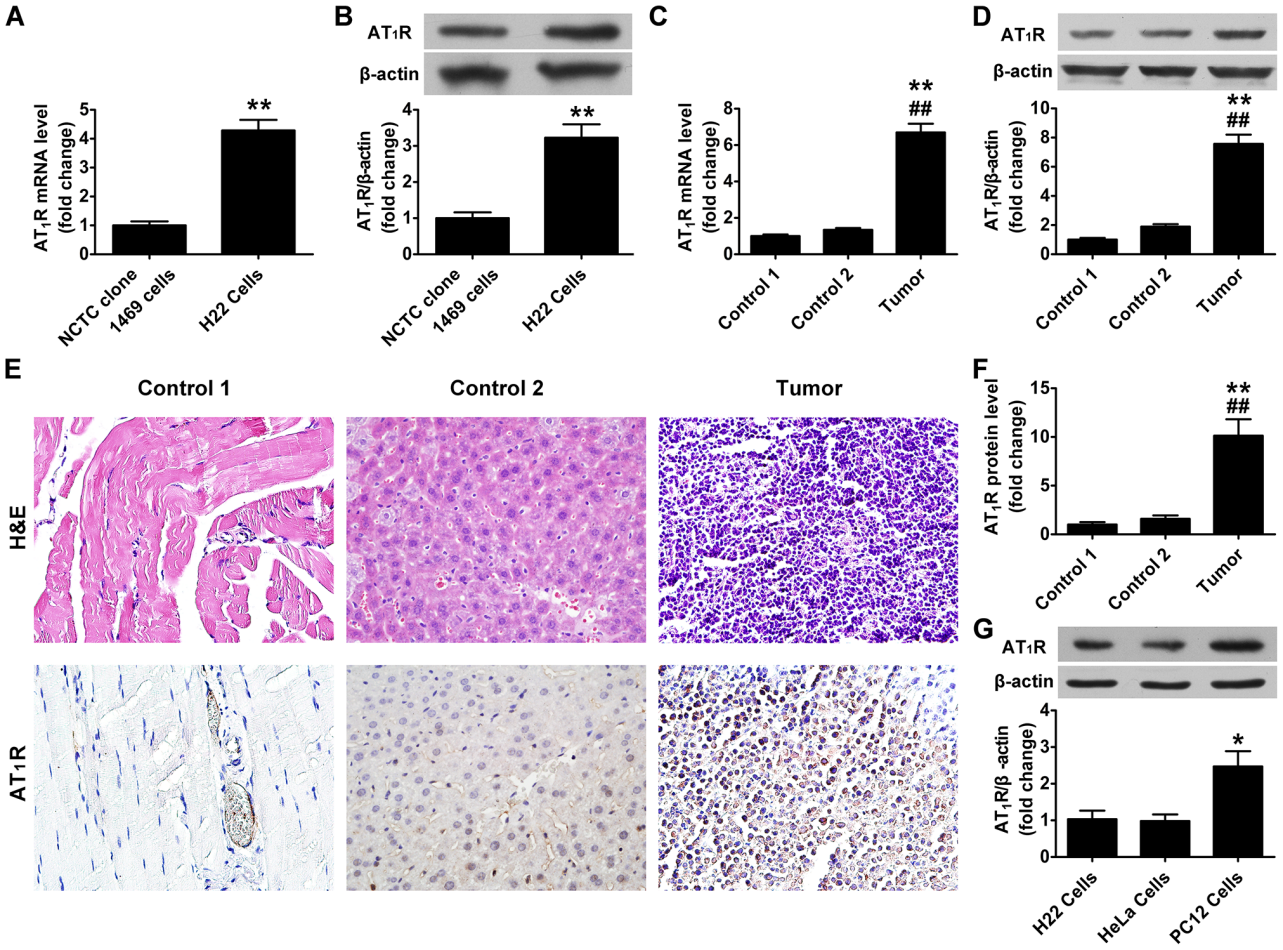
AT_1_R mRNA and protein expression. (A) Relative mRNA expression of AT_1_R in NCTC clone 1469 cells (normal hepatocytes) and H22 cells. **P<0.01 vs. NCTC clone 1469 cells. (B) Representative Western blot of AT_1_R and quantification of AT_1_R protein levels in NCTC clone 1469 cells and H22 cells. **P<0.01 vs. NCTC clone 1469 cells. (C) Relative mRNA expression of AT_1_R in the muscle of the opposite side (control 1), normal liver tissue (control 2) and hepatocellular carcinoma tissue. **P<0.01 vs. control 1. ^##^ *P*<0.01 vs. control 2. (D) Representative Western blot of AT_1_R and quantification of AT_1_R protein levels in control 1, control 2 and hepatocellular carcinoma tissue. **P<0.01 vs. control 1. ^##^ *P*<0.01 vs. control 2. (E) Representative hematoxylin and eosin staining (H&E) and immunohistochemical staining for AT_1_R (original magnification 400×). (F) Quantification of AT_1_R protein levels in immunohistochemical staining. **P<0.01 vs. control 1. ^##^ *P*<0.01 vs. control 2. (G) Representative Western blot of AT_1_R and quantification of AT_1_R protein levels in H22 cells, HeLa cells and PC12 cells. *P<0.05 vs. H22 cells.

AT_1_R mRNA expression was quantified by real-time RT-PCR. There was a markedly higher AT_1_R mRNA level in H22 cells than that in NCTC clone 1469 cells (Fig. 3A). Similarly, there was a significantly higher AT_1_R mRNA level in hepatocellular carcinoma tissue than that in contralateral muscle (control 1) or normal liver tissue (control 2) (Fig. 3C). AT_1_R protein expression was assessed by immunohistochemistry staining and Western blot. AT_1_R protein was mainly localized to cell membranes (Fig. 3E). AT_1_R protein level was significantly higher in H22 cells than that in NCTC clone 1469 cells (Fig. 3B). Similarly, AT_1_R protein level was significantly higher in hepatocellular carcinoma tissue than that in control 1 or control 2 (Fig. 3D, 3F). In addition, AT_1_R protein level was higher in PC12 cells than in H22 or HeLa cells (Fig. 3G).

Attempts have never ceased for diagnosis and surveillance of tumor. Although biopsy is still the gold standard for monitoring and predicting the process of tumor, early protocol biopsies can not be performed conventionally due to its invasive characteristic. Imaging examination becomes an ideal method owing to its noninvasive characteristic and good repeatability. Molecular imaging, which integrates imaging techniques with molecule biology, is more sensitive and can detect abnormalities before the appearance of symptoms and signs. Screening for optimal molecules as biomarkers plays a key role in molecular imaging. An ideal biomarker for tumor should be closely related to tumor progression, and absolutely or relatively specific for diagnosis and surveillance of tumor.

The utility of molecular imaging in hepatocellular carcinoma mainly relies on the non-invasive detection of cytokines or other molecules secreted by the tumor evenly before the functional and structural changes can be discovered [19], [20]. The early detection of tumor could improve survival greatly and the benefits of molecular imaging should not be underestimated. Identification of novel targets and predictors through molecular cell biology will identify new diagnostic strategies for early stage hepatocellular carcinoma and provide better methods for outcome prediction.

The renin-angiotensin system (RAS) plays an important role in tumor growth and angiogenesis [21]–[23]. Ang II, the main effector peptide of RAS, was reported to be involved in the development of several tumors, including breast, ovarian and pancreatic cancers [24]–[26]. Ang II exerts a variety of biological actions through binding to AT_1_R [27]. Thus, interference with AT_1_R by declining the level of Ang II or antagonizing the receptor may impair tumor growth and angiogenesis [28].

This study suggested that AT_1_R was overexpressed in hepatocellular carcinoma tissue using a mouse hepatoma model and revealed that hepatocellular carcinoma tissue could specifically uptake ^131^I-anti-AT_1_R mAb but not ^131^I-IgG. In the early stage of imaging, the two agents were infiltrated in the intercellular space non-specifically through binding to the receptor of fragments crystallizable to IgG on the tissues. In the late stage of imaging, the non-specific accumulation of ^131^I-IgG decreased gradually, while the specific accumulation of ^131^I-anti-AT_1_R mAb increased markedly, attributed to the increased expression of AT_1_R in the tumor.

One limitation of our study should be pointed out. Overexpression of AT_1_R may be present in other kinds of tumors, such as cervical cancer and adrenal pheochromocytoma. Thus, it is difficult to differentiate hepatocellular carcinoma from other kinds of tumors in the liver tissue.

In conclusion, our study indicated that ^131^I-anti-AT_1_R mAb enables non-invasive evaluation of hepatocellular carcinoma specifically and may be a new potential molecular imaging agent for targeting tumor.
